# 2246: Characterization of resistant hypertension in a statewide electronic health record-based database (OneFlorida)

**DOI:** 10.1017/cts.2017.63

**Published:** 2018-05-10

**Authors:** Caitrin W. McDonough, William R. Hogan, Betsy Shenkman, Rhonda M. Cooper-DeHoff

## Abstract

OBJECTIVES/SPECIFIC AIMS: Our objective is to create a Resistant Hypertension (RHTN) computable phenotype from electronic health record (EHR)-based data, and to determine the characteristics associated with RHTN within a large, diverse, EHR-based database. METHODS/STUDY POPULATION: The OneFlorida Clinical Research Consortium includes 10 unique health care systems providing care for approximately half of the state (48%, ~10 million). OneFlorida houses a Data Trust which contains longitudinal EHR data and claims data from these providers in a common format, the PCORnet common data model v3.0. For the current project, data from 5 health care systems were considered. All of the adult hypertension (HTN) patients with a HTN diagnosis from an outpatient encounter were extracted from the OneFlorida Data Trust. Additional data such as demographics, prescribing, and vitals information were also extracted. The RHTN computable phenotype was created by constructing a drug exposure variable that took into consideration the number of antihypertensive medications an individual was prescribed at any point in time over the course of the OneFlorida dataset. RHTN was defined as any blood pressure requiring four or more antihypertensive drugs, or uncontrolled blood pressure (≥140/90) on 3 antihypertensive drugs. RHTN cases had to meet the definition criteria twice during the data period, at least 30 days apart. All data extraction, computation phenotype coding, and statistical analyses were conducted using SQL or SAS. RESULTS/ANTICIPATED RESULTS: Our preliminary results show that there were n=342,026 adults with a HTN diagnosis from an outpatient visit in the data set. After the RHTN computable phenotype was constructed, n=11,670 RHTN cases were identified from the n=130,901 HTN individuals with all of the required variables in the data set (8.9% RHTN prevalence). In all, 55% of RHTN cases were Black or African American, compared with the total HTN population (25% Black/African American). RHTN cases also had more prescriptions for loop diuretics, centrally acting agents, α-blockers, and vasodilators compared with the total HTN population. Not surprisingly, the RHTN cases had 26% of the antihypertensive prescriptions in the data set, and the RHTN cases had fewer blood pressure readings that were in control (only 49.4% of readings <140/90). DISCUSSION/SIGNIFICANCE OF IMPACT: Overall, our preliminary data shows that it is possible to create the very complicated computable phenotype of RHTN within the OneFlorida Data Trust. We found that the RHTN prevalence in OneFlorida is 8.9% which is consistent with previous studies from NHANES. Although promising, these results require further validation of the computable phenotype and replication in other similar data sets in order to ascertain their true meaning. Once validated, the experience gained from this computable phenotype can be applied to many other phenotypes.

